# Double-Cavity Fabry–Perot Interferometer Sensor Based on Polymer-Filled Hollow Core Fiber for Simultaneous Measurement of Temperature and Gas Pressure

**DOI:** 10.3390/s25082396

**Published:** 2025-04-10

**Authors:** Yixin Zhu, Yufeng Zhang, Qianhao Tang, Shengjie Li, Huaijin Zheng, Dezhi Liang, Haibing Xiao, Chenlin Du, Yongqin Yu, Shuangchen Ruan

**Affiliations:** 1 Key Laboratory of Advanced Optical Precision Manufacturing Technology of Guangdong Higher Education Institutes, Sino-German College of Intelligent Manufacturing, Shenzhen Technology University, Shenzhen 518118, China; 2210412023@stumail.sztu.edu.cn (Y.Z.); 2210412014@stumail.sztu.edu.cn (Q.T.); 2310412032@stumail.sztu.edu.cn (S.L.); 2310412028@stumail.sztu.edu.cn (H.Z.); ruanshuangchen@sztu.edu.cn (S.R.); 2 Physics Teaching and Experiment Center, Shenzhen Technology University, Shenzhen 518118, China; zhangyufeng@sztu.edu.cn (Y.Z.); liangdezhi@sztu.edu.cn (D.L.); 3 Intelligent Manufacturing and Equipment School, Shenzhen Institute of Information Technology, Shenzhen 518172, China; xiaohb@sziit.edu.cn

**Keywords:** double-polymer cavities, hollow core fiber, optical fiber sensors, temperature and gas pressure measurement, high sensitivity

## Abstract

A double-cavity Fabry-Perot (F-P) interferometer sensor based on a polymer-filled hollow core fiber (HCF) has been proposed and experimentally verified. The double cavity of the sensor is formed by filling the hollow core fiber with two kinds of polymer materials and curing these materials, with the other end of the hollow core fiber connected to a single-mode fiber (SMF). The three reflective surfaces of the sensor reflect three beams of light, which interfere to form a spectrum with an envelope. By using Fast Fourier Transform (FFT) and a Fourier filter, the spectrum of each cavity can be separated and, based on this, the demodulation matrix of the sensor can be constructed. By controlling the length of the polymer cavity, a single sensor cavity can achieve high temperature and gas pressure sensitivity, with values of 2.05 nm/°C and 17.63 nm/MPa, respectively. More importantly, the sensor can be used under an environment of 40–110 °C and 0–3.0 MPa, with simple fabrication, good robustness, and better stability and repeatability compared to similar sensors. Based on its high sensitivity and large measurement range, this sensor has broad application prospects in industrial manufacturing and harsh environmental monitoring fields.

## 1. Introduction

Optical fiber sensors have been widely used in industrial production, environmental monitoring, biomedicine, and other fields due to their small size, light weight, resistance to electromagnetic interference, and corrosion resistance. Temperature and gas pressure are important physical quantities, and there are currently various optical fiber sensors used for monitoring temperature and gas pressure detection, including fiber Bragg gratings (FBGs), long-period fiber Bragg gratings (LPFGs), Mach–Zehnder (MZ) interferometers, Fabry–Perot interferometers, whispering gallery mode (WGM) resonant microcavities, etc. [1,2,3,4,5,6,7]. Among them, optical fiber F-P interferometer sensors have always been a key focus of research in optical fiber sensing applications due to their simple structure, high reliability, and low manufacturing cost.

F-P interferometers based on all-fiber structures have low sensitivities due to the limitations of the low thermal expansion coefficient (TEC) and low thermal optical coefficient (TOC) of the silicon dioxide material itself. By using materials with higher TOCs and TECs, such as mercury, polyacrylate, glycerol, polydimethylsiloxane, etc., for the manufacturing of optical fiber sensors, the temperature or gas pressure sensitivity of the sensor can be significantly improved [8,9,10,11,12,13,14,15,16]. These F-P interferometers have high sensitivities to both temperature and gas pressure. One way to eliminate cross-sensitivity between temperature and gas pressure is to connect an FBG in front of the F-P interferometer and track the spectral changes in the FBG to determine the variables of the surrounding environment temperature, thereby eliminating cross-sensitivity [15,16,17,18]. Another method is to construct multiple reflection surfaces to form multi-beam interference and use multiple F-P cavities to construct a matrix to eliminate the cross-sensitivity of temperature and gas pressure. Multiple sensors with two F-P cavities have been proposed, most of which are based on an air cavity and a material-filled cavity [10,19,20,21]. Due to the existence of air cavities, most of these sensors have a high sensitivity to temperature and gas pressure. However, the structural strength of the air cavity is relatively low, and in order to prevent damage to the sensor and ensure the stability of the sensing measurement, the gas pressure measurement range of the sensor is limited.

In this article, we propose a high-sensitivity F-P cavity sensor for temperature and gas pressure measurement. This sensor is made of two types of polymer materials filled with HCF and can perform double-parameter sensing in the temperature range of 40–110 °C and the gas pressure range of 0–3.0 MPa. The temperature and gas pressure sensitivities can reach 2.05 nm/°C and 17.63 nm/MPa, respectively, and it has high stability and repeatability. By constructing a demodulation matrix, cross-sensitivity between temperature and gas pressure can be eliminated. We also study the relationship between the filling length and sensitivity of two types of polymers in the sensor. The experimental results showed that the filling length of the polymer was positively correlated with the sensitivity, but increasing the filling length of the polymer would lead to a decrease in the stability of the sensor. Therefore, it is necessary to make sensors with appropriate structural dimensions according to actual needs. Based on these characteristics, our proposed sensor has broad application prospects in industrial manufacturing and harsh environmental monitoring fields.

## 2. Fabrication and Principle

### 2.1. Sensor Fabrication

**Figure 1:**
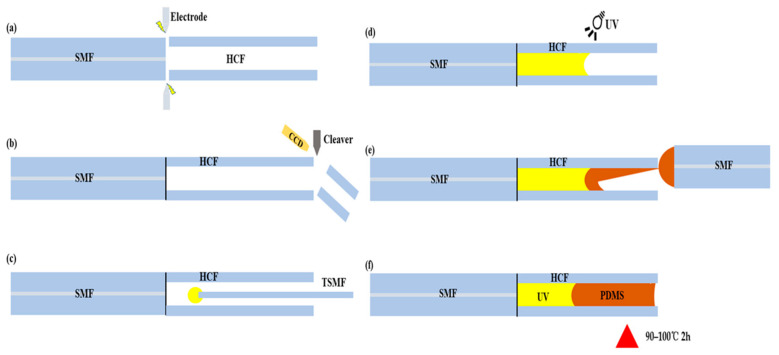
Sensor manufacturing process diagram. (**a**) Spliced SMF with HCF. (**b**) Retain the HCF of a specific length. (**c**) Using TSMF to feed the UV glue into the bottom of the HCF. (**d**) The UV glue is cured by irradiating with UV lamp. (**e**) The process of offset filling PDMS. (**f**) The heat curing process of the PDMS.

The production process of this sensor mainly consisted of the following six steps: the fusion splicing of the HCF, cutting and retaining specific lengths of HCF, filling ultraviolet (UV) glue into the HCF using a tapered single-mode fiber (TSMF), the UV curing of the UV glue (9300, LEAFTOP, Shenzhen, China), the offset filling of polydimethylsiloxane (PDMS) glue, and the heat curing of the PDMS glue. The manufacturing process of the sensor is shown in Figure 1. The following describes the manufacturing procedure: Firstly, take a single-mode fiber (SMF-28, Corning, New York, NY, USA) with a core diameter of 8.2 μm and a cladding diameter of 125 μm and use fiber stripping pliers to remove its coating layer. Take a hollow core fiber (TSP075150, Polymicro Technologies, Phoenix, AZ, USA) with an inner diameter of 75 μm and a cladding outer diameter of 125 μm. Use a blade to remove the polyimide coating on its surface and use a cutting knife (FC-6, Sumitomo Electric, Osaka, Japan) to cut the fiber’s end faces flat. Then, use a fusion splicer (FSM-100M+, Fujikura, Tokyo, Japan) to fuse the two fibers together. The second step is to place the fused SMF-HCF on a cutting blade, observe and measure the distance from the fusion point to the cutting blade by using a charge-coupled device (CCD) camera, adjust the position of the fiber, and cut it to retain a specific length of HCF. For step three, use a fusion splicer to taper an SMF and obtain a tapered fiber with a diameter of 35 μm. Use a sapphire blade to cut the tapered fiber from the tapered waist to obtain a relatively flat tapered fiber end face. Dip the end face of the TSMF vertically into a small amount of UV glue on a glass slide, and use a coupling alignment platform (Apico, Shenzhen, China) to feed the UV glue into the bottom of the HCF. Repeat this process several times. Step four, use a UV lamp to irradiate the filled UV glue for about 20 min to ensure the complete curing of the UV glue. Step five, mix the PDMS base agent (DC184-A, Dow Corning, Midland, TX, USA) with the curing agent (DC184-B) in a mass ratio of 10:1 and let it stand for 30 min until all the bubbles produced during the mixing process disappear completely, obtaining a PDMS solution. Vertically dip the end face of another SMF that has been cut flat into the PDMS solution on a glass slide and perform offset filling on the platform. Due to capillary effect, the PDMS solution will gradually flow into the inner wall of the HCF and expel the air. Repeat this offset filling multiple times until the HCF is completely filled. Finally, place the sensor in a drying oven (220-00A, Kun Tian, Shanghai, China) and heat it at 90–100 °C for 2 h to fully cure the PDMS adhesive.

**Figure 2:**
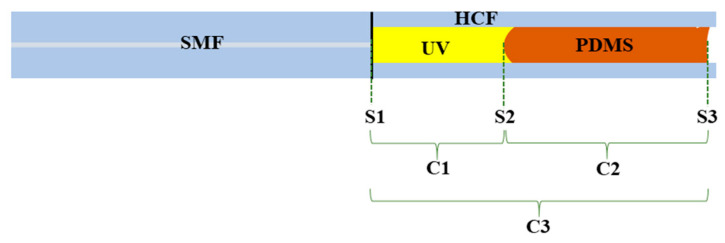
Schematic diagram of sensor structure.

The manufactured sensors were mainly divided into three F-P cavities, namely C1, C2, and C3. Cavity C1 was the part filled with UV glue, from the splicing end face (S1) of the SMF and HCF to the interface between the UV glue and PDMS solution (S2). The C2 cavity was the part filled with PDMS solution, from S2 to the interface between the PDMS solution and air (S3). The C3 cavity was constituted by S1 and S3, and in terms of measurement length, C3 = C1 + C2. The sensor structure is shown in Figure 2. Since there are three reflective surfaces, S1, S2, and S3, when light reaches each of these three reflective surfaces, reflections will occur. There is an optical path difference (OPD) between the reflected lights, thus forming three-beam interference.

### 2.2. Sensor Principle

**Figure 3:**
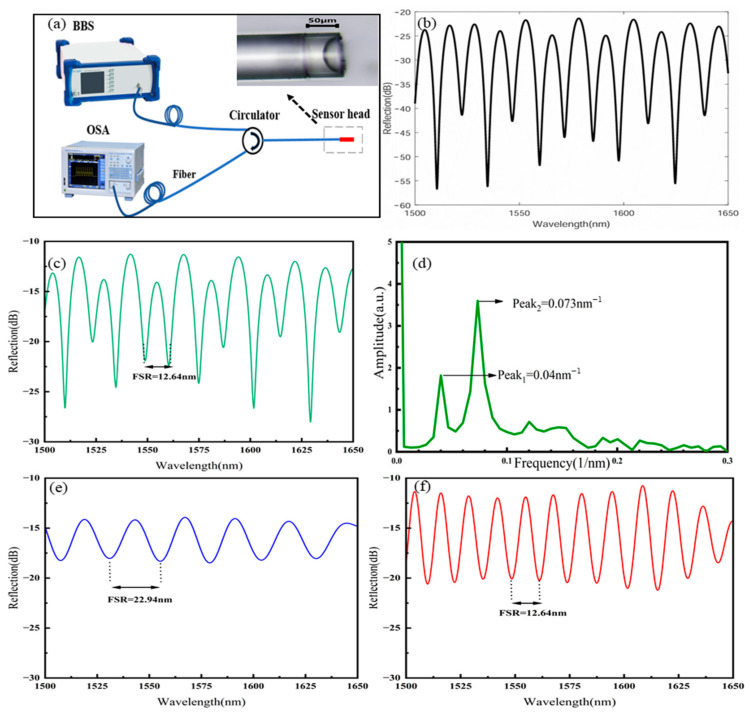
(**a**) A schematic diagram of the structure used for measuring the sensor’s spectra. (**b**) The theoretical simulation spectrum. (**c**) The measured interference spectrum of the sensor (at room temperature and standard atmospheric pressure). (**d**) The FFT spectrum. (**e**) The separated spectrum of cavity C1. (**f**) The separated spectrum of cavity C3.

A spectrometer (AQ6370D, Yokogawa, Tokyo, Japan) (OSA) and a broadband light source (Broadband Light Source, Golight Technology, Shenzhen, China) (BBS) were connected to the sensor probe through a circulator to measure the reflected spectrum of the sensor probe, as shown in Figure 3a. The small figure in Figure 3a shows the microscopic image of Sample 1 sensor. The lengths of cavity C1 and cavity C2 in Sample 1 were 36.0 μm and 30.7 μm, respectively. The refractive index of C1 after UV glue curing was n_1_ = 1.48, and the refractive index of C3 after PDMS solution curing was n_2_ = 1.40. We used MATLAB (2018a) to simulate the spectrum of Sample 1 based on the cavity length measured by the microscope and the known refractive index of the material, as shown in Figure 3b. Figure 3c shows the three-beam interference spectrum of the Sample 1 sensor that was measured by a spectrometer. The measured spectrum was consistent with the theoretical simulated spectrum, which indicated that three-beam interference indeed occurred within the sensor. The OPD of the interference beams within each cavity were OPD_1_ = 2n_1_L_1_, OPD_2_ = 2n_2_L_2_, and OPD_3_ = 2(n_1_L_1_ + n_2_L_2_). According to Formula (1), the free spectral ranges (FSRs) of each cavity spectrum could be calculated (λ taken as 1550 nm) as FSR_1_ = 22.55 nm, FSR_2_ = 27.95 nm, and FSR_3_ = 12.48 nm. According to Formula (2), the frequencies corresponding to each cavity were determined to be ƒ_1_ = 0.044 nm^−1^, ƒ_2_ = 0.036 nm^−1^, and ƒ_3_ = 0.080 nm^−1^. We performed FFT on the interference spectrum (the wavelength range selected for all FFTs in the paper was 1500 nm to 1650 nm) of the sensor to obtain the FFT spectrum that is shown in Figure 3d. Two distinct frequency peaks could be observed in the figure, located at 0.04 nm^−1^ and 0.073 nm^−1^ respectively, corresponding to the theoretically calculated ƒ_1_ and ƒ_3_. This indicated that the low-frequency interference in the spectrum mainly originated from cavity C1, while the high-frequency interference in the spectrum came from cavity C3.

The relationship between free spectral range and optical path difference can be calculated as follows:

(1) $$FSR=\frac{\lambda^{2}}{\mathrm{OPD}}$$

The relationship between frequency ƒ after FFT and free spectral range can be calculated as follows:

(2) $$ƒ=\frac{1}{FSR}$$

Based on the FFT spectrum shown in Figure 3d, the interference spectrum was filtered using an FFT low-pass filter (cut-off frequency: 0.055 nm^−1^) and an FFT band-pass filter (lower cut-off frequency: 0.060 nm^−1^; upper cut-off frequency: 0.096 nm^−1^) to separate the spectra of cavity C1 and cavity C3, as shown in Figure 3e,f. From the separated spectra of the C1 and C3 cavities, the free spectral ranges could be directly read as FSR1 = 22.94 nm and FSR2 = 12.64 nm, which were close to the theoretically calculated FSR values for cavities C1 and C3. This indicated that the separated spectra of the C1 and C3 cavities obtained through FFT filtering were essentially consistent with the actual spectra of the C1 and C3 cavities. (For more analysis about why C2 does not appear in the FFT plot and why low-pass and band-pass filters are used, please refer to the Supplementary Materials).

Cavities C1 and C3 were filled with UV adhesive and PDMS adhesive, respectively, and these two adhesives have different TECs, TOCs, and Shore hardnesses (SHs), which are as follows: TEC_UV_ = 2.75 * 10^−4^/°C; TEC_PDMS_ = 9.6 * 10^−4^/°C; TOC_UV_ = 1.82 * 10^−4^/°C; TOC_PDMS_ = −4.5 * 10^−4^/°C; SH_UV_ = 70 HD; and SH_PDMS_ = 43 HA. Therefore, when the surrounding environmental temperature or gas pressure changed, the refractive indices and lengths of the UV adhesive and PDMS adhesive filling the two cavities changed differently. This resulted in different sensitivities of the UV adhesive and PDMS adhesive to temperature and gas pressure, ultimately leading to different spectral wavelength shifts in cavities C1 and C3.

The wavelength shifts in the separated spectra of cavities C1 and C3 can be expressed as follows:

(3) $$\left(\begin{array}{c} ∆\lambda_{1}\\ ∆\lambda_{3} \end{array}\right)=\left(\begin{array}{cc} K_{1,P} & K_{1,T}\\ K_{3,P} & K_{3,T} \end{array}\right)\left(\begin{array}{c} ∆P\\ ∆T \end{array}\right)$$

Here, Δ*λ*_1_ and Δ*λ*_3_ represent the wavelength shift changes in the spectra in cavities C1 and C3, respectively. *K*_1,*P*_ and *K*_3,*P*_ represent the gas pressure sensitivities of cavities C1 and C3, respectively. *K*_1,*T*_ and *K*_3,*T*_ represent the temperature sensitivities of cavities C1 and C3, respectively. Δ*P* and Δ*T* represent the changes in the surrounding environmental gas pressure and temperature, respectively. *K*_1,*P*_, *K*_3,*P*_, *K*_1,*T*_, and *K*_3,*T*_ can be obtained by calculating the responses of the separated spectra of cavities C1 and C3 to temperature and gas pressure, respectively. Therefore, by solving the matrix, the changes in the surrounding environmental temperature and gas pressure can be expressed as follows:

(4) $$\left(\begin{array}{c} ∆P\\ ∆T \end{array}\right)=\frac{1}{K_{1,P}K_{3,T}-K_{1,T}K_{3,P}}\left(\begin{array}{cc} K_{3,T} & -K_{1,T}\\ -K_{3,P} & K_{1,P} \end{array}\right)\left(\begin{array}{c} ∆\lambda_{1}\\ ∆\lambda_{3} \end{array}\right)$$

Using this method, the changes in the surrounding environmental temperature and gas pressure could be solved through the wavelength changes in the separated spectra of cavities C1 and C3. This approach not only achieved double-parameter sensing but also eliminated the impact of cross-sensitivity through matrix solving.

## 3. Experimental Setup

**Figure 4:**
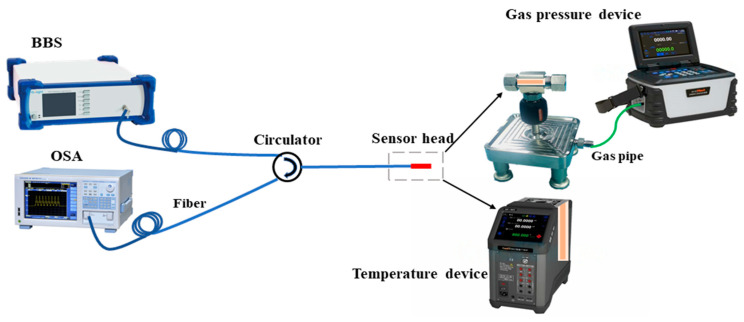
Schematic diagram of temperature and gas pressure experimental setup.

The experimental setup for measuring the temperature and gas pressure responses of the sensor samples is shown in Figure 4. The sensor probe was inserted into a temperature-controlled oven (ConST660, ConST Instruments Technology, Beijing, China), and the temperature inside the oven was set to increase from 40 °C to 110 °C in steps of 5 °C. After the oven reached each set temperature, it was maintained for 10 min, during which time the changes in the sensor’s reflected spectrum were recorded. The sensor probe was also inserted into a gas pressure cavity, which was connected to a pressure pump (ConST811, ConST Instruments Technology, Beijing, China) through a gas pipeline. The gas pressure inside the cavity was changed by injecting or releasing gas through the pressure pump. When the gas pressure inside the cavity was equal to the atmospheric pressure outside, it was recorded as 0 MPa. The gas pressure was increased from 0 MPa to 1.0 MPa in steps of 0.1 MPa, and after the cavity reached each set pressure it was maintained for 5 min, during which time the changes in the sensor’s reflected spectrum were recorded. It should be noted that the temperature of the compressed air in the pressure pump was different from the initial air temperature in the 0 MPa pressure cavity. Therefore, when the pressure pump started to inject compressed air into the 0 MPa pressure cavity, the temperature of the air inside the cavity changed significantly. To minimize the impact of the temperature change in the compressed air in the pressure cavity on the gas pressure response, the gas pressure data in the range of 0–0.2 MPa were discarded when analyzing the spectral changes.

## 4. Results and Discussion

### 4.1. Relationship Between Sensor Cavity Length and Sensitivity

To investigate the relationship between the polymer cavity length and the temperature and gas pressure sensitivity of the sensor, we designed and fabricated three sensors of different dimensions; the specific lengths of their polymer cavities and the frequencies calculated based on the cavity lengths are shown in Table 1:

Temperature and gas pressure response experiments were conducted on the three sensors. The interference spectra of the sensors were transformed using Fast Fourier Transform and, based on the FFT spectra, FFT low-pass and band-pass filtering were applied to obtain the separated spectra of cavities C1 and C3 as they varied with temperature and gas pressure. Linear fitting was performed on the resonance peak shifts in the separated spectra of the C1 and C3 cavities with temperature and gas pressure to determine the temperature and gas pressure sensitivities of these cavities. Finally, the relationship between the lengths of the C1 and C3 cavities and their sensitivities was analyzed.

#### 4.1.1. Sensor 1 Temperature and Gas Pressure Responses

**Figure 5:**
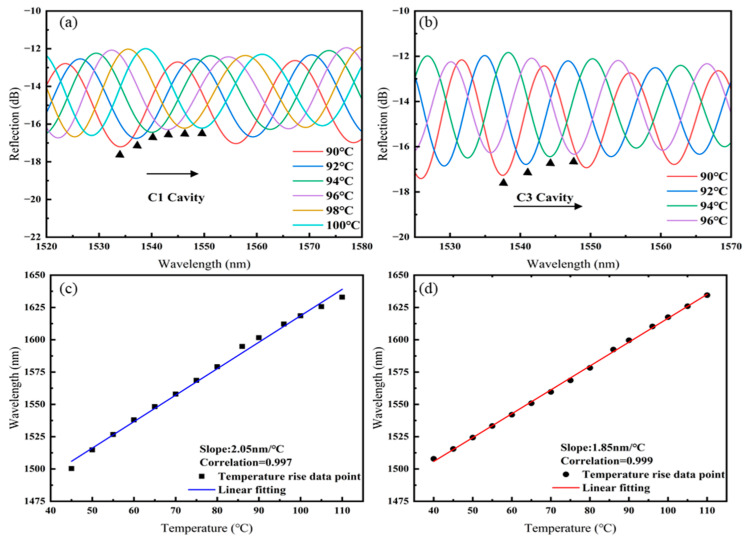
Sensor 1: (**a**) Spectral shift in C1 with increasing temperature, (**b**) spectral shift in C3 with increasing temperature, (**c**) linear fitting of temperature sensitivity for C1, (**d**) linear fitting of temperature sensitivity for C3.

The interference spectrum of Sensor 1 was filtered using an FFT low-pass filter (cut-off frequency: 0.055 nm^−1^) and an FFT band-pass filter (lower cut-off frequency: 0.060 nm^−1^; upper cut-off frequency: 0.096 nm^−1^) to separate the spectra of cavity C1 and cavity C3 as they vary with temperature, as shown in Figure 5a,b. As the temperature increases, the spectra of both the C1 and C3 cavities shift towards longer wavelengths. The linear fitting of the temperature responses of cavities C1 and C3 of Sensor 1, as shown in Figure 5c,d, results in temperature sensitivities of 2.05 nm/°C for C1 and 1.85 nm/°C for C3.

**Figure 6:**
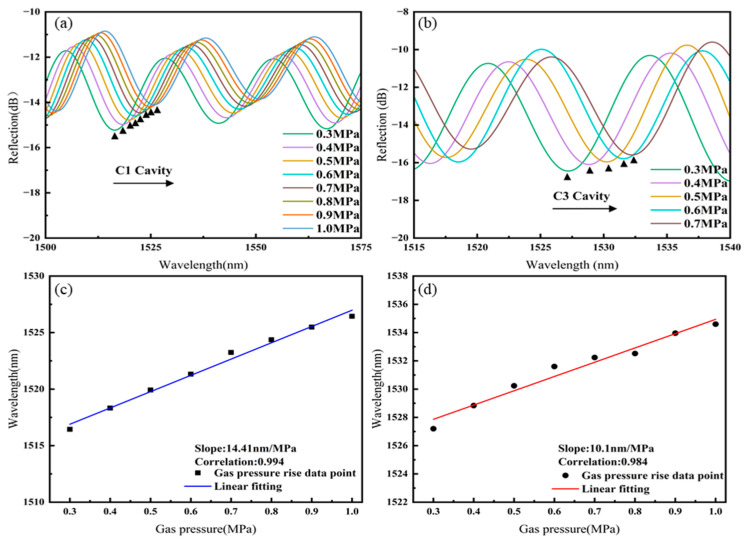
Sensor 1: (**a**) Spectral shift in C1 with increasing gas pressure, (**b**) spectral shift in C3 with increasing gas pressure, (**c**) linear fitting of gas pressure sensitivity for C1, (**d**) linear fitting of gas pressure sensitivity for C3.

The separated spectra of cavities C1 and C3 of Sensor 1 as they vary with gas pressure are shown in Figure 6a,b. As the gas pressure increases, the spectra of both C1 and C3 cavities shift towards longer wavelengths. The linear fitting of the gas pressure responses of cavities C1 and C3 of Sensor 1, as shown in Figure 6c,d, results in gas pressure sensitivities of 14.41 nm/MPa for C1 and 10.1 nm/MPa for C3.

#### 4.1.2. Sensor 2 Temperature and Gas Pressure Responses

**Figure 7:**
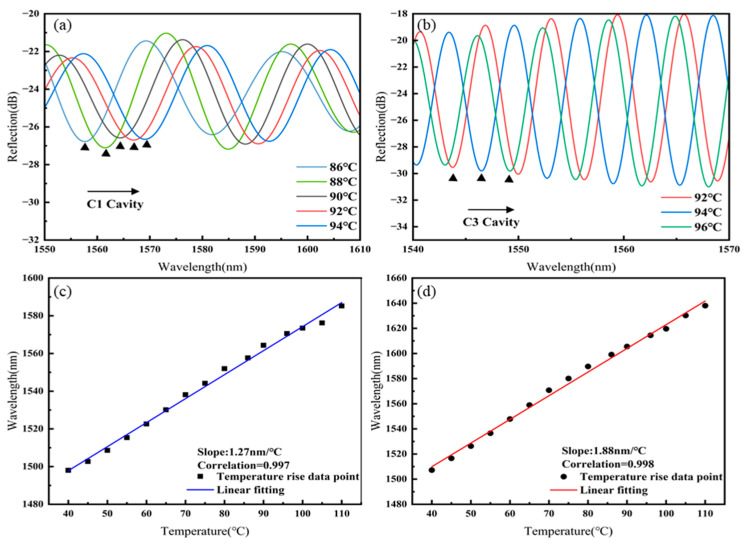
Sensor 2: (**a**) Spectral shift in C1 with increasing temperature, (**b**) spectral shift in C3 with increasing temperature, (**c**) linear fitting of temperature sensitivity for C1, (**d**) linear fitting of temperature sensitivity for C3.

The interference spectrum of Sensor 2 was filtered using an FFT low-pass filter (cut-off frequency: 0.065 nm^−1^) and an FFT band-pass filter (lower cut-off frequency: 0.122 nm^−1^; upper cut-off frequency: 0.170 nm^−1^) to separate the spectra of cavity C1 and cavity C3 as they vary with temperature, as shown in Figure 7a,b. As the temperature increases, the spectra of both the C1 and C3 cavities shift towards longer wavelengths. The linear fitting of the temperature responses of cavities C1 and C3 of Sensor 2, as shown in Figure 7c,d, results in temperature sensitivities of 1.27 nm/°C for C1 and 1.88 nm/°C for C3.

**Figure 8:**
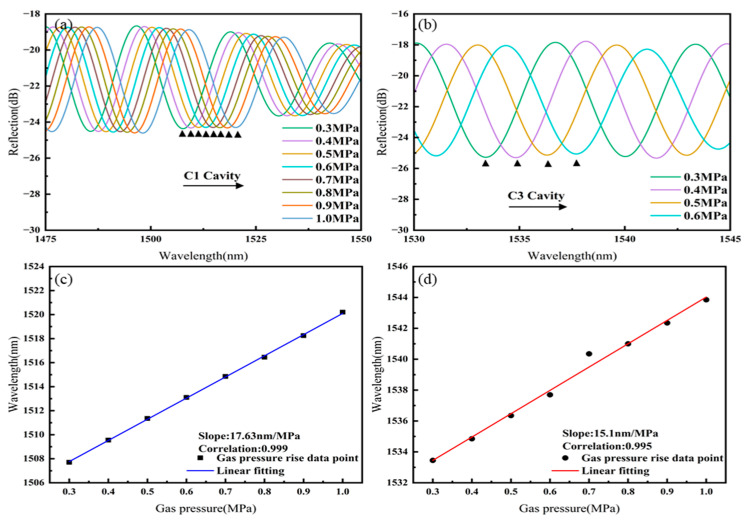
Sensor 2: (**a**) Spectral shift in C1 with increasing gas pressure, (**b**) spectral shift in C3 with increasing gas pressure, (**c**) linear fitting of gas pressure sensitivity for C1, (**d**) linear fitting of gas pressure sensitivity for C3.

The separated spectra of cavities C1 and C3 of Sensor 2 as they vary with gas pressure are shown in Figure 8a,b. As the gas pressure increases, the spectra of both C1 and C3 cavities shift towards longer wavelengths. The linear fitting of the gas pressure responses of cavities C1 and C3 of Sensor 2, as shown in Figure 8c,d, results in gas pressure sensitivities of 17.63 nm/MPa for C1 and 15.1 nm/MPa for C3.

#### 4.1.3. Sensor 3 Temperature and Gas Pressure Responses

**Figure 9:**
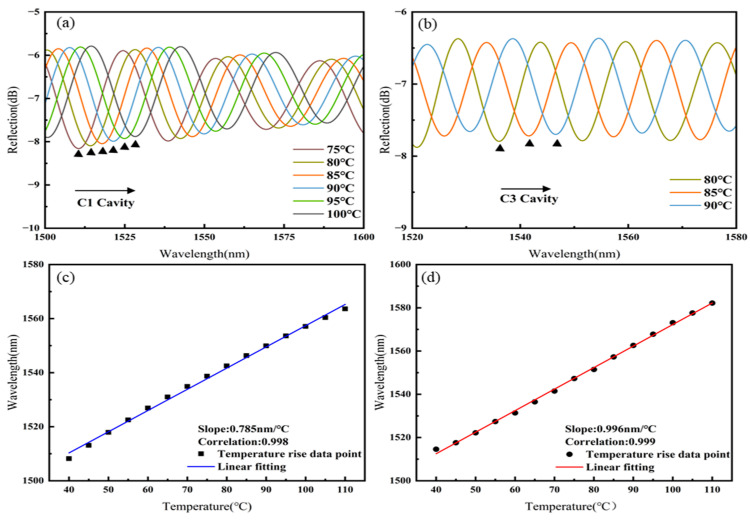
Sensor 3: (**a**) Spectral shift in C1 with increasing temperature, (**b**) spectral shift in C3 with increasing temperature, (**c**) linear fitting of temperature sensitivity for C1, (**d**) linear fitting of temperature sensitivity for C3.

The interference spectrum of Sensor 3 was filtered using an FFT low-pass filter (cut-off frequency: 0.040 nm^−1^) and an FFT band-pass filter (lower cut-off frequency: 0.053 nm^−1^; upper cut-off frequency: 0.070 nm^−1^), as shown in Figure 9a,b. As the temperature increases, the spectra of both C1 and C3 cavities shift towards longer wavelengths. The linear fitting of the temperature responses of cavities C1 and C3 of Sensor 3, as shown in Figure 9c,d, results in temperature sensitivities of 0.785 nm/°C for C1 and 0.996 nm/°C for C3.

**Figure 10:**
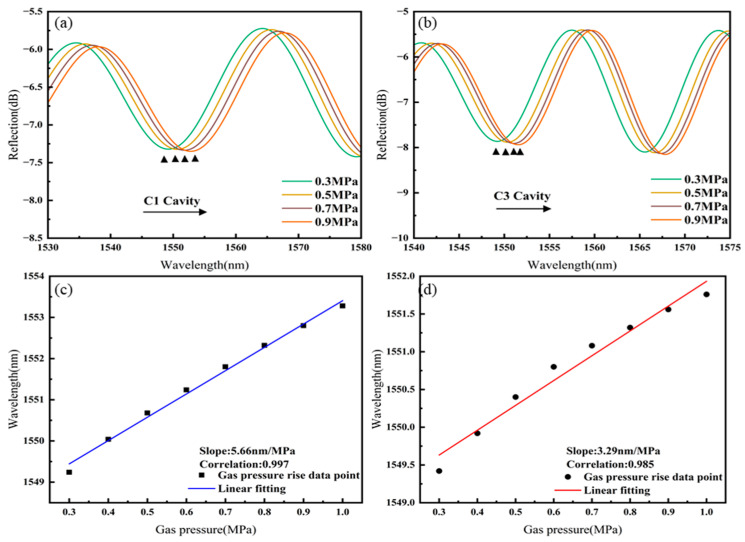
Sensor 3: (**a**) Spectral shift in C1 with increasing gas pressure, (**b**) spectral shift in C3 with increasing gas pressure, (**c**) linear fitting of gas pressure sensitivity for C1, (**d**) linear fitting of gas pressure sensitivity for C3.

The separated spectra of cavities C1 and C3 of Sensor 3 as they vary with gas pressure are shown in Figure 10a,b. As the gas pressure increases, the spectra of both C1 and C3 cavities shift towards longer wavelengths. The linear fitting of the gas pressure responses of cavities C1 and C3 of Sensor 3, as shown in Figure 10c,d, results in gas pressure sensitivities of 5.66 nm/MPa for C1 and 3.29 nm/MPa for C3.

According to Equation (4), the parameters in the demodulation matrices of the three samples can be determined. The demodulation matrix for Sensor 1 is as follows:

(5) $$\left(\begin{array}{c} ∆P\\ ∆T \end{array}\right)=0.168\left(\begin{array}{cc} 1.85 & -2.05\\ -10.1 & 14.41 \end{array}\right)\left(\begin{array}{c} ∆\lambda_{1}\\ ∆\lambda_{3} \end{array}\right)$$

The demodulation matrix for Sensor 2 is as follows:

(6) $$\left(\begin{array}{c} ∆P\\ ∆T \end{array}\right)=0.0716\left(\begin{array}{cc} 1.88 & -1.27\\ -15.1 & 17.63 \end{array}\right)\left(\begin{array}{c} ∆\lambda_{1}\\ ∆\lambda_{3} \end{array}\right)$$

The demodulation matrix for Sensor 3 is as follows:

(7) $$\left(\begin{array}{c} ∆P\\ ∆T \end{array}\right)=0.327\left(\begin{array}{cc} 0.996 & -0.785\\ -3.29 & 5.66 \end{array}\right)\left(\begin{array}{c} ∆\lambda_{1}\\ ∆\lambda_{3} \end{array}\right)$$

Table 2 shows the relationship between the lengths of the polymer cavities and the temperature and gas pressure sensitivities. Overall, as the length of the polymer cavity increases, more polymer is filled, and the temperature and gas pressure sensitivities of the corresponding cavity both increase. However, the relationship between cavity length and sensitivity is only positively correlated, not linear. Moreover, the C1 cavity length and gas pressure sensitivity of Samples 1 and 2 show a negative correlation. We suggest that this is because the curvature at interface S2 between the UV glue and the PDMS adhesive is different for the two samples. Sensor 2 has a deeper concavity on the S2 surface (concavity about 38 μm) compared to Sensor 1’s concavity on the S2 surface (concavity about 21 μm), which results in a greater curvature. Combining our findings with existing research [22], when the interface has a greater concavity and curvature, the temperature sensitivity of the sensor increases. Therefore, we suggest that when the interface has a greater concavity and curvature, the pressure sensitivity of the sensor may also increase. Meanwhile, the C1 cavity lengths of Sensor 1 and 2 are close, making them more susceptible to the influence of the curvature at S2, ultimately leading to a negative correlation between the C1 cavity length and gas pressure sensitivity of the two samples. Overall, the cavity length of the sensor is positively correlated with its temperature and pressure sensitivity, although this conclusion is susceptible to the influence of the interface curvature when the cavity lengths are similar.

The above analyses comprehensively indicate that increasing the length of the polymer cavity can enhance the temperature and gas pressure sensitivity of the sensor by retaining a longer portion of HCF and being able to fill it with more UV glue and PDMS polymer. Then, by observing the length of the polymer-filled cavity under a microscope and repeatedly dipping the cavity into TSMF to adjust the cavity length, it is possible to achieve controllable polymer cavity length in sensor fabrication. However, according to Equation (1), an increase in cavity length also leads to a decrease in free spectral range, which is detrimental to spectral tracking. Moreover, to achieve a longer polymer cavity, a longer segment of HCF must be retained during the sensor fabrication process. This not only makes the operation shown in Figure 1c more challenging but also results in a larger curvature at interface S2 due to the surface tension of the UV glue itself, which is not conducive to controlling the sensor’s sensitivity.

### 4.2. Stability Testing of Sensors

To measure the impact of fluctuations due to sensor stability on temperature and gas pressure measurements, we roughly calculated the overall spectral sensitivity of the three sensors to temperature and gas pressure. These sensitivity data were only used for analyzing sensor stability and were not meaningful for our matrix calculations. The overall spectral sensitivities of Sensors 1–3 to temperature changes are 1.89 nm/°C, 1.53 nm/°C, and 0.91 nm/°C, respectively; the overall spectral sensitivities of Sensors 1–3 to gas pressure changes are 12.41 nm/MPa, 15.95 nm/MPa, and 3.99 nm/MPa, respectively.

**Figure 11:**
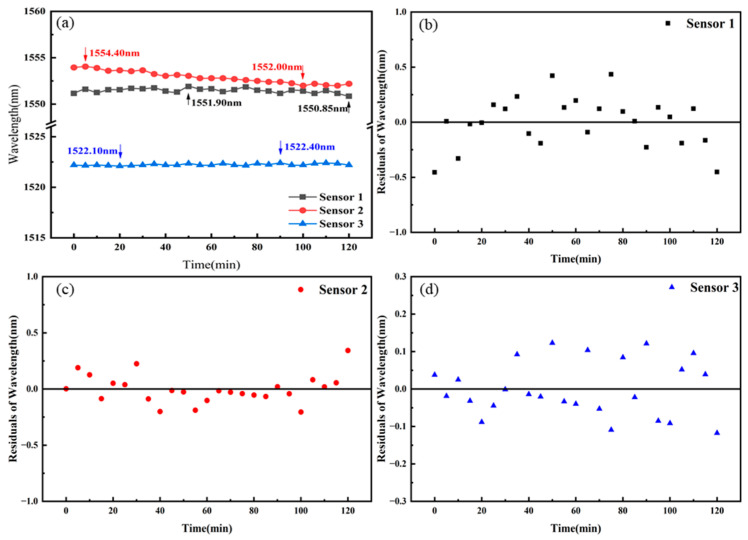
(**a**) Temperature stability tests of Sensors 1–3. (**b**) Residual plot of Sensor 1. (**c**) Residual plot of Sensor 2. (**d**) Residual plot of Sensor 3.

We measured the temperature stability of Sensors 1–3 at 70 °C, as shown in Figure 11a. Each of the three sensors was placed in a temperature-controlled oven set to maintain 70 °C. We monitored a specific peak position in the overall interference spectra of the sensors, recording the peak position changes every 5 min over a period of 120 min. For Sensor 1, the maximum and minimum peak positions were 1551.90 nm and 1550.85 nm, respectively, with a maximum fluctuation of 1.05 nm. The residual plot of Sensor 1 is shown in Figure 11b, with the data points randomly distributed within the range of ±0.5 nm. For Sensor 2, the maximum and minimum peak positions were 1554.05 nm and 1552.00 nm, respectively, with a maximum fluctuation of 2.05 nm. The residual plot of Sensor 2 is shown in Figure 11c, with the data points randomly distributed within the range of ±0.5 nm. For Sensor 3, the maximum and minimum peak positions were 1552.40 nm and 1552.10 nm, respectively, with a maximum fluctuation of 0.30 nm. The residual plot of Sensor 3 is shown in Figure 11d, with the data points randomly distributed within the range of ±0.2 nm. The corresponding temperature fluctuations for the maximum spectral fluctuations were 0.56 °C, 1.34 °C, and 0.32 °C, respectively.

**Figure 12:**
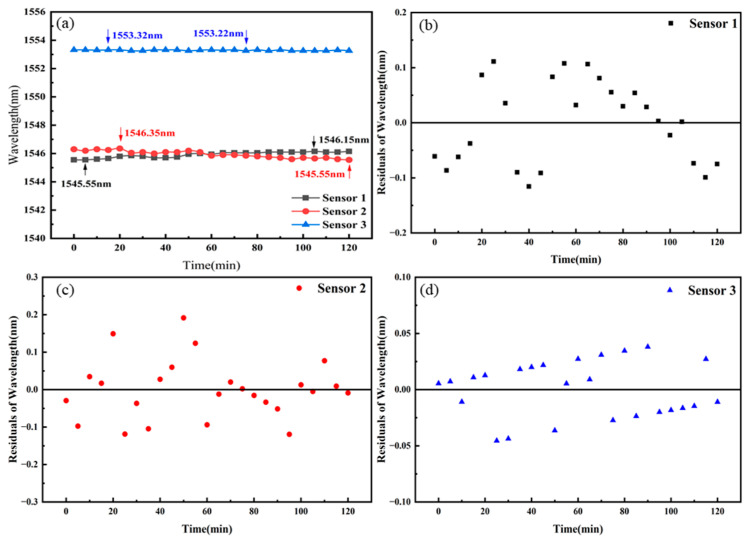
(**a**) Gas pressure stability tests of Sensors 1–3. (**b**) Residual plot of Sensor 1. (**c**) Residual plot of Sensor 2. (**d**) Residual plot of Sensor 3.

We also measured the gas pressure stability of the three sensors at 0.5 MPa, as shown in Figure 12a. Each of the three sensors was placed in a gas pressure cavity, and the gas pressure pump was set to maintain the gas pressure in the cavity at 0.5 MPa. We monitored a specific peak position in the overall interference spectrum of the sensors, recording the peak position changes every 5 min over a period of 120 min. For Sensor 1, the maximum and minimum peak positions were 1546.15 nm and 1545.55 nm, respectively, with a maximum fluctuation of 0.60 nm. The residual plot of Sensor 1 is shown in Figure 12b, with the data points randomly distributed within the range of ±0.15 nm. For Sensor 2, the maximum and minimum peak positions were 1556.35 nm and 1545.55 nm, respectively, with a maximum fluctuation of 0.80 nm. The residual plot of Sensor 2 is shown in Figure 12c, with the data points randomly distributed within the range of ±0.2 nm. For Sensor 3, the maximum and minimum peak positions were 1552.40 nm and 1552.10 nm, respectively, with a maximum fluctuation of 0.10 nm. The residual plot of Sensor 3 is shown in Figure 12d, with the data points randomly distributed within the range of ±0.05 nm. The corresponding gas pressure fluctuations for the maximum spectral fluctuations were 0.048 MPa, 0.050 MPa, and 0.025 MPa, respectively.

The maximum fluctuation ranges corresponding to temperature and gas pressure for the three samples under the set conditions are very small. The residual plot also shows that the wavelength fluctuates randomly within a small range and does not vary with changes in temperature and gas pressure. These results indicate good temperature and gas pressure stability for all three samples. Moreover, Sensor 3 shows significantly smaller temperature and gas pressure fluctuations compared to Sensors 1 and 2, demonstrating better stability. This suggests that, when the length of the polymer cavity in the sensor increases, the temperature and gas pressure sensitivities are enhanced, but this is accompanied by a certain degree of reduced stability.

### 4.3. High-Gas-Pressure Response Test of Sensors

To verify the high-gas-pressure performance limits of the sensors, we additionally fabricated Sensor 4. Considering the studies on sensor sensitivity and stability discussed above, the dimensions of Sample 4 were designed to be similar to those of Sensor 3. The C1 cavity of Sample 4 was 24.1 μm long, the C2 cavity was 29.3 μm long, and the C3 cavity was 53.4 μm long. Using the same method as for Samples 1–3, we measured the gas pressure sensitivities of the C1 and C3 cavities of Sample 4 under 0–1.0 MPa (low-gas-pressure range) to be 1.32 nm/MPa and 2.39 nm/MPa, respectively. Subsequently, we increased the gas pressure from 1.0 MPa to 3.0 MPa (high-gas-pressure range) in steps of 0.5 MPa, maintaining each gas pressure setting in the cavity for 3 min before recording the spectral changes.

**Figure 13:**
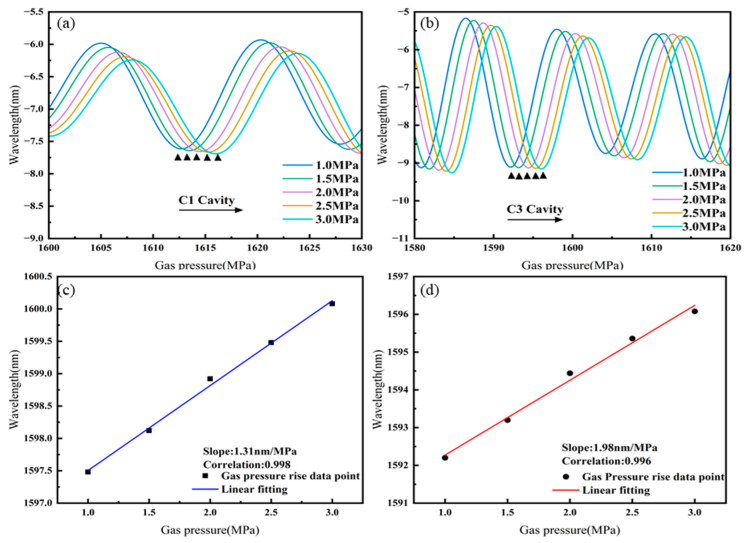
Sensor 4: (**a**) Spectral shift in C1 with increasing gas pressure, (**b**) spectral shift in C3 with increasing gas pressure, (**c**) linear fitting of gas pressure sensitivity for C1, (**d**) linear fitting of gas pressure sensitivity for C3.

The separated spectra of cavities C1 and C3 of Sensor 4 as they vary with gas pressure in the high-gas-pressure range are shown in Figure 13a,b. As the gas pressure increases, the spectra of both C1 and C3 cavities shift towards longer wavelengths. The linear fitting of the gas pressure responses of cavities C1 and C3 of Sensor 4, as shown in Figure 13c,d, shows gas pressure sensitivities of 1.31 nm/MPa for C1 and 1.98 nm/MPa for C3 in the high-gas-pressure range.

**Figure 14:**
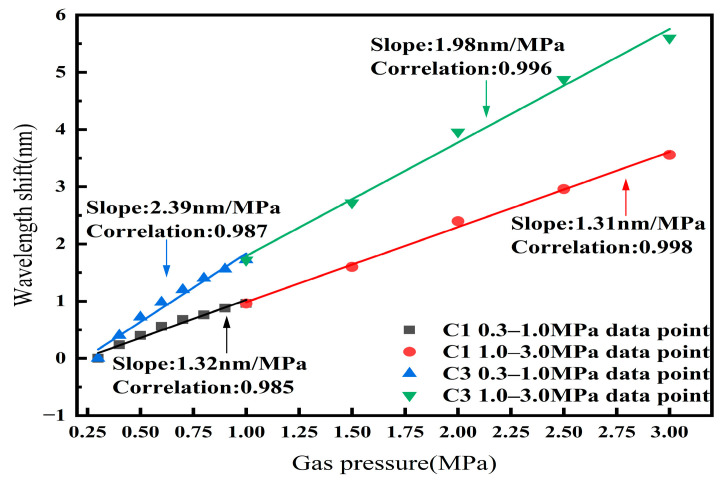
Linear fitting plots of C1 and C3 of sensor in the 0–1.0 MPa and 1.0–3.0 MPa ranges.

The linear fitting plots of the two cavities of Sensor 4 in the 0–1.0 MPa range and the 1.0–3.0 MPa range were drawn on the same graph, as shown in Figure 14. The linear fitting plots of cavity C1 and cavity C3 in the high-gas-pressure range can be seen as an extension of the linear fitting plots in the low-gas-pressure range, and the gas pressure sensitivities in the high-gas-pressure and low-gas-pressure ranges are similar. This demonstrates that the effective gas pressure sensing range of the designed sensor is 0–3.0 MPa. This greatly expands the gas pressure detection range of the sensor, making the designed sensor applicable to more scenarios.

### 4.4. Repeatability Testing of Sensors

**Figure 15:**
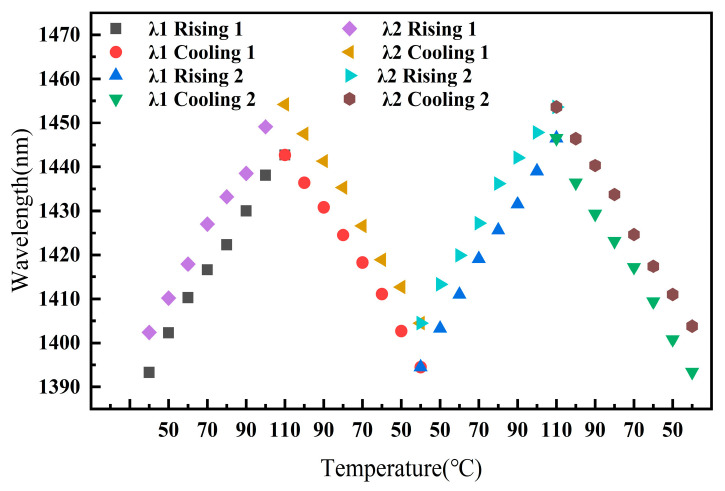
The verification of the temperature repeatability of the sensor.

To verify the repeatability of the sensor’s readings, Sample 4 was placed in a temperature-controlled oven, and the oven was set to increase in temperature from 40 °C to 110 °C in steps of 10 °C and then decrease its temperature back to 40 °C in the same steps, repeating this cycle twice. During this process, the changes in the peak positions of the overall sensor spectrum were recorded. Figure 15 shows the changes in the positions of two different peak wavelengths, λ1 and λ2, during the temperature cycling process. After two cycles of temperature increase and decrease, the final positions of λ1 and λ2 changed by 0.1 nm and 1.4 nm, respectively, compared to their initial positions. These changes are relatively small compared to the overall spectral changes within the measurement range of the sensor, indicating the good temperature repeatability of the sensor.

**Figure 16:**
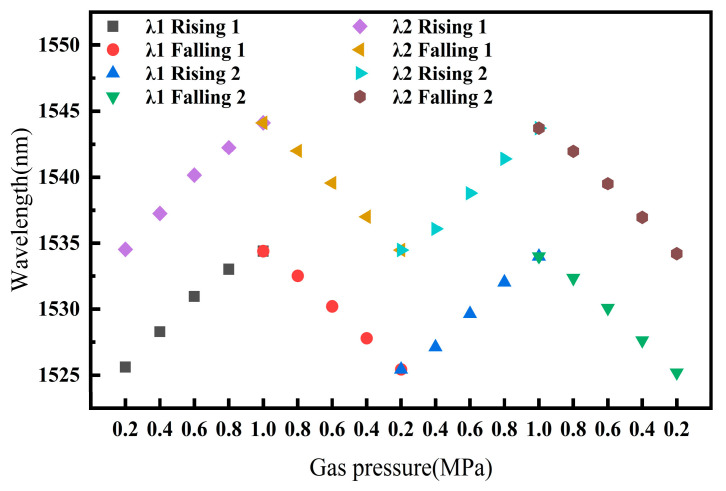
Gas pressure repeatability experiment for sensor.

In the same manner, the gas pressure repeatability experiment of the sensor was conducted. Sample 4 was placed in a gas pressure cavity, and the gas pressure inside the cavity was controlled by a gas pressure pump to increase in steps of 0.2 MPa up to 1.0 MPa and then decrease back to 0.2 MPa, repeating this cycle twice. During this process, the changes in the peak positions of the overall sensor spectrum were recorded. Figure 16 shows the changes in the positions of two different peak wavelengths, λ1 and λ2, during the gas pressure cycling process. After two cycles of gas pressure increase and decrease, the final positions of λ1 and λ2 changed by 0.42 nm and 0.32 nm, respectively, compared to their initial positions. These changes are relatively small compared to the overall spectral changes within the measurement range of the sensor, indicating the good gas pressure repeatability of the sensor.

Table 3 shows the temperature and gas pressure sensing performance of several different types of sensors. The temperature and gas pressure sensitivities of the sensor we fabricated are higher than those of most sensors in Table 3. Compared to the sensors with higher gas pressure sensitivities than ours, such as reference [23], the sensor we fabricated can operate at a broader range of gas pressures. This is because sensor structures that use thin films or retain air cavities improve their sensitivity with the trade-off of a reduced gas pressure sensing range and structural strength. A broader gas pressure measurement range is one of the significant achievements of our work. In addition, our sensor is capable of matrix demodulation, which many of the sensors in Table 3 do not possess. However, we found that sensors made from polymer materials are limited by the inherent temperature tolerance of the polymer itself, restricting their temperature sensing range.

## 5. Conclusions

In summary, based on the F-P cavity multi-beam interference principle, we designed and fabricated an F-P type sensor using a double-cavity polymer-filled hollow core fiber with PDMS and UV adhesives. We employed FFT filters to separate the interference spectra and measured the temperature and gas pressure sensitivities of the two cavities, constructing a matrix for the sensor in order to achieve double-parameter sensing of temperature and gas pressure, thereby resolving the issue of cross-sensitivity. This sensor is capable of double-parameter sensing within a temperature range of 40–110 °C and a gas pressure range of 0–3.0 MPa, with high temperature and gas pressure sensitivities (2.05 nm/°C, 17.63 nm/MPa), and it exhibits excellent stability and repeatability. The fabrication cost is low, and the sensor is robust. By controlling the lengths of the two polymer cavities during sensor fabrication, sensitivity adjustment can be achieved. Given these features, the designed sensor holds broad application prospects in the fields of industrial manufacturing and harsh environment monitoring.

## Figures and Tables

**Table 1:** Dimensions and frequencies of sensors with different polymer cavity lengths.

Sample Number	C1 Length (μm)	C1 Frequency (nm^−1^)	C2 Length (μm)	C2 Frequency (nm^−1^)	C3 Length (μm)	C3 Frequency (nm^−1^)
1	36.0	0.044	30.7	0.036	66.7	0.080
2	35.3	0.043	85.2	0.099	120.5	0.143
3	24.6	0.030	29.4	0.034	54.0	0.065

**Table 2:** Relationship between different polymer cavity lengths and temperature and gas pressure sensitivities.

Sample Number	C1 Length (μm)	C1 Temperature Sensitivity (nm/°C)	C1 Gas Pressure Sensitivity (nm/MPa)	C3 Length (μm)	C3 Temperature Sensitivity (nm/°C)	C3 Gas Pressure Sensitivity (nm/MPa)
1	36.0	2.05	14.41	66.7	1.85	10.10
2	35.3	1.27	17.63	120.5	1.88	15.10
3	24.6	0.785	5.66	54.0	0.996	3.29

**Table 3:** Comparison of temperature and gas pressure sensing performance of different sensors.

Type	Max. Temperature Sensitivity (nm/°C)	Temperature Sensing Range (°C)	Max. Gas Pressure Sensitivity (nm/MPa)	Gas Pressure Sensing Range (MPa)	Reference
FBG + F-P	0.748	30–70	8.45	0.1–0.7	[15]
F-P with photopolymer material	−1.18	20–110	_	_	[24]
LPFG coated with PDMS	−0.484	30–60	−18.26	0–0.4	[12]
Twin core fiber with M-Z	0.043	10–100	8.45	0–2.0	[25]
All-fiber cavity F-P	0.01083	25–300	4.1587	0–0.8	[26]
Fiber-tip with UV	0.249	40–90	1.13	0.1–2.5	[27]
Fiber-tip with two materials	0.68968	20–75	_	_	[28]
HCF with PDMS film	0.131	20–50	52.143	0.1–0.7	[23]
Double-cavity with two materials	2.05	40–110	17.63	0.3–3.0	This work

## Data Availability

Data are contained within the article.

## Supplementary Materials

The following supporting information can be downloaded at: https://www.mdpi.com/article/10.3390/s25082396/s1, Figure S1: FFT spectrum of Sensor 2.
