# Supplementary material for: Double-Cavity Fabry–Perot Interferometer Sensor Based on Polymer-Filled Hollow Core Fiber for Simultaneous Measurement of Temperature and Gas Pressure
Source: Sensors (Basel). 2025 Apr 10;25(8):2396. doi: 10.3390/s25082396 (PMC12031117; doi:10.3390/s25082396)
Supplement: Supplementary file 1 [file sensors-25-02396-s001.zip › sensors-3554785-supplementary.pdf]

Supplementary

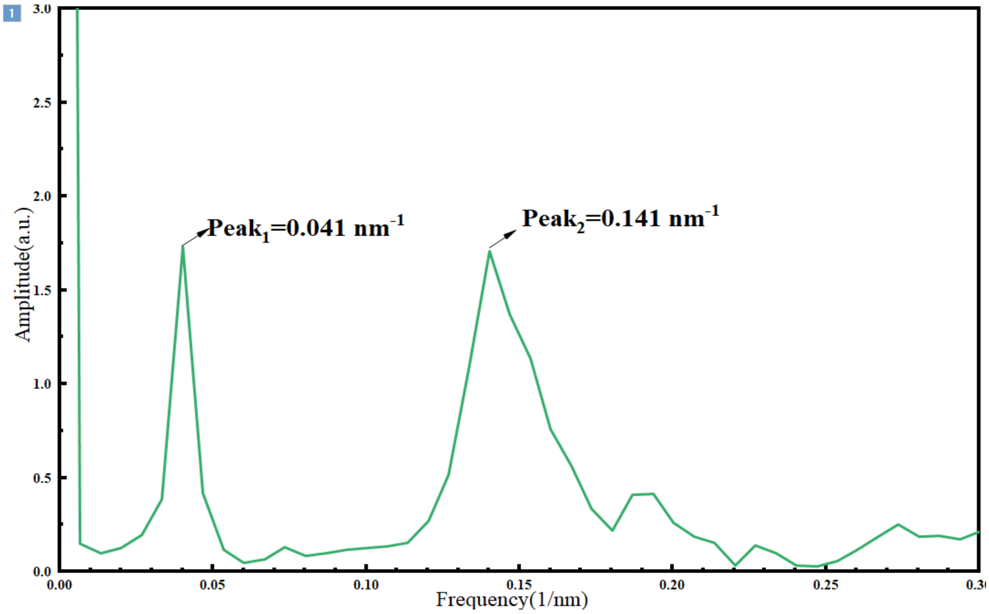

Supplementary Figure S1. FFT spectrum of Sensor 2.

For Sensor 2 (L_1=35.3 μm, L_2=85.2 μm, n_1=1.48, n_2=1.40), the frequencies corresponding to different cavities can be calculated.

$$FSR = \frac{\lambda^2}{\text{OPD}}$$

$$OPD_1 = 2n_1L_1,\ OPD_2 = 2n_2L_2,\ OPD_3 = 2(n_1L_1 + n_2L_2)$$

$$f_1 = \frac{1}{FSR_1} = 0.043nm^{-1},\ f_2 = \frac{1}{FSR_3} = 0.099nm^{-1},\ f_3 = \frac{1}{FSR_3} = 0.143nm^{-1}$$

From the **Supplementary Figure S1.**, two peaks can be observed (f_{peak1}=0.041 nm^{-1}, f_{peak2}=0.141 nm^{-1}), which correspond to the theoretically calculated frequency for cavity C1 (f_1=0.043 nm^{-1}) and C3 (f_3=0.143 nm^{-1}) respectively. Since cavities C1 and C3 are the dominant cavities in generating beam interference, and cavity C2 is not visible in FFT spectrum and cannot be separated by filtering, our work mainly focuses on the study of cavities C1 and C3.

In the process of filtering the peaks corresponding to cavities C1 and C3 to obtain the separated spectra, we used a low-pass filter and a band-pass filter, respectively. It is also feasible to use two band-pass filters with different cutoff frequencies. However, a low-pass filter is essentially a special type of band-pass filter with a lower cutoff frequency of 0. Additionally, there are no minor noise peaks to the left of peak1. Therefore, we decided to use a low-pass filter to separate the spectrum of cavity C1.
